# Sialic Acid-Functionalized Gold Nanoparticles for Sensitive and Selective Colorimetric Determination of Serotonin

**DOI:** 10.1021/acsomega.4c01859

**Published:** 2024-05-23

**Authors:** Begüm Avcı, Yeliz Akpınar, Gülay Ertaş, Mürvet Volkan

**Affiliations:** † Department of Chemistry, Middle East Technical University, 06800 Ankara, Turkey; ‡ Department of Chemistry, Kirsehir Ahi Evran University, 40100 Kirsehir, Turkey

## Abstract

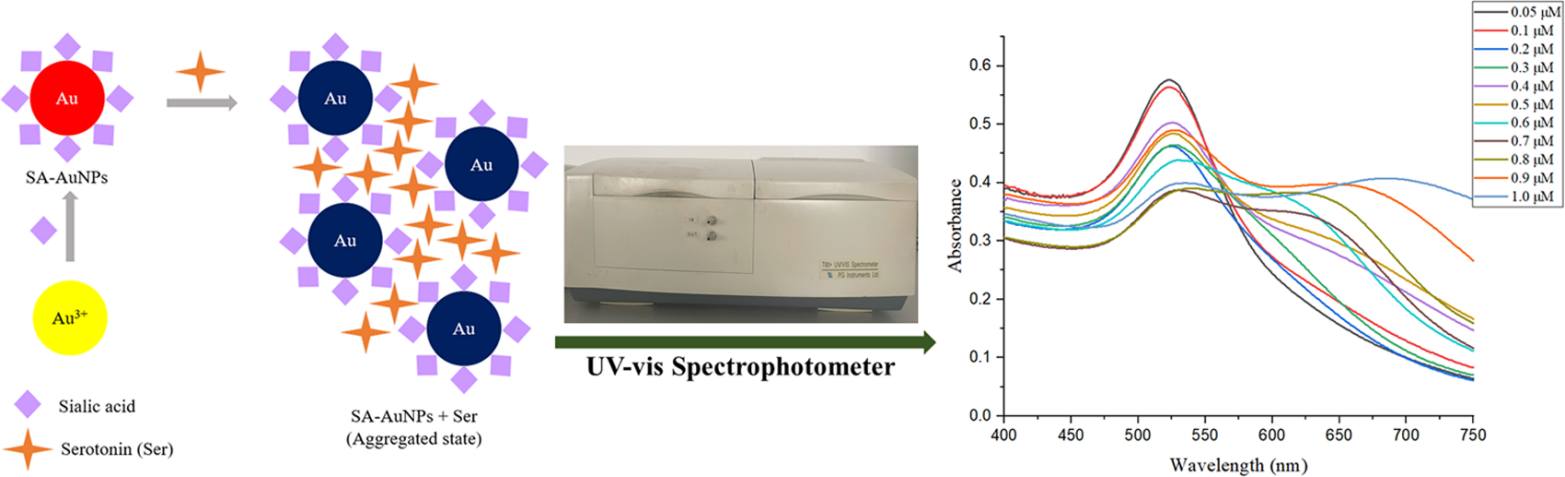

We present a novel colorimetric method inspired by nature’s complex mechanisms, capable of selectively determining serotonin with high sensitivity. This method exploits the inherent binding affinity of serotonin with sialic acid (SA) molecules anchored to gold nanoparticles (SA-AuNPs). Upon serotonin binding, SA-AuNPs aggregate, and a characteristic red shift in the absorbance of SA-AuNPs accompanied by a dramatic color change (red to blue) occurs, readily observable even without instrumentation. The proposed method effectively eliminates interventions from potential interfering species such as dopamine, epinephrine, l-tyrosine, glucosamine, galactose, mannose, and oxalic acid. The absence of a color change with l-tryptophan, a structurally related precursor of serotonin, further confirms the high selectivity of this approach for serotonin detection. The colorimetric method has a wide linear dynamic range (0.05–1.0 μM), low limit of detection (0.02 μM), and fast response time (5 min). The limit of detection of the method is lower than other colorimetric serotonin sensors reported so far. The possible use of the proposed method in biological sample analysis was evaluated by employing a serotonin recovery assay in processed human plasma. The recoveries ranged from 90.5 to 104.2%, showing promising potential for clinical applications.

## 1 Introduction

Serotonin (5-hydroxytryptamine, 5-HT) is a small and important molecule that has two functions in the human body: a monoamine type of neurotransmitter in the central nervous system and a hormone in the periphery.^1^ It possesses a highly important role in regulating many behavioral and cognitive functions like mood,^2^ sleep,^3^ appetites,^4^ learning,^5^ pain,^6^ sexuality,^7^ and cardiovascular function.^8^ Serotonin levels in human blood are between 0.6 and 1.6 μM for healthy people.^9^ High and low levels of serotonin have been linked to several diseases. Its low levels have been associated with depression and anxiety,^10^ migraine,^11^ attention deficit hyperactivity disorder (ADHD),^12^ and inflammatory syndromes,^13^ whereas high levels are associated with serotonin toxicity or syndrome^14^ and carcinoid tumors.^15,16^ The relationship between serotonin levels and specific diseases evaluates serotonin levels as a tool for diagnosis. For this purpose, several methods have been developed to determine serotonin including mass spectrometry,^17−19^ electrochemical techniques,^20−25^ phosphorescence,^26^ fluorimetry,^27−32^ and surface-enhanced Raman spectrometry.^33,34^ Although these methods can successfully measure low serotonin levels, most of them require high-cost equipment, and additionally, they are time-consuming, particularly methods involving liquid chromatography.

Colorimetric assays using chromogenic probes that change color in response to a specific target have gained popularity in analytical applications due to their simplicity and affordability. Chen and co-workers developed a selective chromogenic probe for the determination of nitrite in foods based on the reaction between nitrite and the amino group of 3,3,5,5-tetramethylbenzidine (TMB) to produce a diazonium salt. Following that, the diazonium salt reacts with glucosamine hydrochloride to produce an orange molecule determined by the colorimetric method.^35^ In addition, Fernandes and co-workers were able to analyze H_2_S contamination in water by using the red luminescent ruthenium (Ru)-imidazophenanthroline complex as a probe.^36^ Gold nanoparticles, in particular, are becoming a popular choice for designing chromogenic probes due to their unique characteristic called localized surface plasmon resonance (LSPR). This property arises from the collective oscillation of their conducting electrons when they are exposed to electromagnetic radiation. As a result, they strongly absorb or scatter radiation at specific wavelengths, which are determined by the size, shape, composition, interparticle distance, and refractive index of their surrounding medium. A decrease in the interparticle distance causes a red shift in the LSPR band(s) due to strong overlap between plasmon fields, increasing in intensity, and a noticeable change in the solution’s color. Due to this distinct structural feature, their solutions appear colored in the visible spectrum. The color depends on how closely packed the nanoparticles are, with well-separated nanoparticles giving a red color and clustered ones giving a blue color. By engineering the interaction between the nanoparticles’ surface and the analyte, a color shift can be generated. This shift, which is often mediated by electrostatic forces, hydrogen bonds, or even chemical reactions, allows for the visual detection of the analyte.^37−39^ Detection methods developed in recent years based on the LSPR phenomenon are very sensitive and can be used in the determination of different analytes like pesticides,^40^ amino acids,^41^ cancer cells,^42^ and metabolites.^43^

Developing a method for the determination of serotonin in the biological environment is challenging due to both the complexity of the molecule and the interference effects of the matrix. Gold nanoparticles can be used to design a highly accurate chromogenic probe for detecting serotonin. Godoy-Reyes and co-workers developed a probe by functionalizing gold nanoparticles with dithiobis(succinimidyl propionate) (DSP) and *N*-acetyl-l-cysteine (NALC) to increase the selectivity of the functionalized nanoparticles for serotonin, having 0.1 μM limit of detection (LOD).^44^ Chávez and co-workers designed aptamer-gold nanoparticle conjugates to determine serotonin. Although the method displayed good selectivity and sensitivity, it is complicated and expensive.^45^ Another spectrophotometric method for the determination of serotonin derivatives was reported by Jin and co-workers.^46^ The method was based on the formation of a colored product formed from the reaction of serotonin derivatives with *p*-dimethylamino benzaldehyde having an absorbance at 625 nm. The disadvantage of the method was that it was not selective to serotonin and had a relatively high detection limit for serotonin derivatives.

The key to creating an effective chromogenic probe lies in selecting the right molecule to coat the gold nanoparticles. This coating ensures that the probe specifically interacts with serotonin and detects it with a high sensitivity. Sialic acid is involved in many processes of glycans, glycoproteins, and glycolipids and has recognition for many biomolecules by forming specific binding sides,^47^ and one of these biological entities is serotonin.^48^ Therefore, it is a very promising candidate molecule to functionalize gold nanoparticles for the selective determination of serotonin. Sialic acid represents the family of nine-carbon sugar neuraminic acid derivatives. The most common form of sialic acid is the one whose amino group is acetylated, called *N*-acetylneuraminic acid (Neu5Ac).^49^

The nature of the sialic acid interaction with serotonin has been investigated using a range of sialic acids, some derivatives, and analogues.^48,50−52^ An affinity chromatography study by Sturgeon and Sturgeon showed that the presence of acetyl group and the side chain formed from C-7, C-8, and C-9 in sialic acid are necessary for binding of serotonin with high affinity.^51^ For further investigation, Berry and co-workers studied the interaction between serotonin and free sialic acid molecules in an aqueous medium and modeled a noncovalent complex of 5-HT-NeuAc formed from ionic attractions between −COO^–^ and NH_3_^+^ groups by using proton NMR.^53^ Despite numerous experiments, the actual mechanism behind the specific relationship between sialic acid and serotonin has not been understood to date. It is important to conduct further research to reveal the complexities underlying this phenomenon. On the other hand, based on current findings, it appears that the connection between sialic acid and serotonin is established primarily through hydrogen bonding rather than covalent bonding. The *N*-acetyl group, C7−C9 side chain, as well as positive and negative charges on the amine and carboxylic acid groups of sialic acid, contribute to the high-affinity binding of serotonin. Additionally, multiple interaction sites suggest a chelate-type interaction between sialic acid and serotonin, emphasizing the specificity and strength of the sialic acid–serotonin complex.

This specific relationship between sialic acid and serotonin is used to purify some glycoproteins^54^ and to analyze sialo-oligosaccharides and gangliosides,^55^ total *N*-glycans in the cancer cell membrane,^56^ for specific enrichment of Neu5Ac-containing glycopeptides,^47^ and separation of erythropoietin glycoforms.^57^ However, no publication has demonstrated the use of strong and selective sialic acid–serotonin interaction to determine serotonin. Therefore, we developed a colorimetric serotonin sensor that uses chromogenic gold nanoparticles with sialic acid functional groups (SA-AuNPs) synthesized by using *N*-acetylneuraminic acid as a reducing reagent. The sensor detects the aggregation of SA-AuNPs in the presence of serotonin, which acts as a bridge between the gold nanoparticles coated with sialic acid. This binding, through hydrogen bonds, causes the SA-AuNPs to clump together, resulting in a change in the color of the solution. The amount of serotonin in samples like human plasma can be measured by analyzing the color change caused by the aggregation of SA-AuNPs.

## 2 Experimental Details

### 2.1 Materials and Reagents

Gold(III) chloride trihydrate (HAuCl_4_·3H_2_O, ≥99.9% trace metals basis), *N*-acetylneuraminic acid (synthetic, ≥95%), sodium hydroxide (NaOH, puriss., 98–100.5%, pellets), hydrochloric acid (HCl, ACS reagent, 37%), phosphoric acid (85 wt % in H_2_O), sodium chloride (ACS reagent, ≥99.0%), d-(+)-glucosamine hydrochloride (≥99%, crystalline), d-(+)-mannose (synthetic, ≥99%), d-(+)-galactose (≥99%), oxalic acid (98%), l-tyrosine (reagent grade, ≥98% (HPLC)), l-tryptophan, sodium bitartrate (98%), bovine serum albumin, methanol (suitable for HPLC, ≥99.9%), potassium chloride (ACS reagent, 99.0–100.5%), sodium phosphate dibasic (ReagentPlus, ≥99.0%), potassium phosphate monobasic (ACS reagent, ≥99.0%), and silica gel (Davisil grade 644, pore size 150 Å, 100–200 mesh) were purchased from Sigma-Aldrich. Potassium bromide (KBr for IR spectroscopy), sodium dihydrogen phosphate dihydrate (Reag. Ph Eur), ethanol (absolute for analysis), (−)-epinephrine (+)-bitartrate salt, and acetonitrile (gradient grade for liquid chromatography) were purchased from Merck; dopamine hydrochloride was purchased from Sigma; serotonin hydrochloride (98%) was purchased from Alfa Aesar. Zirconyl nitrate (purified) was purchased from Fisher Scientific. Human Plasma Pooled S4180 was purchased from Biowest. Syringe filters (regenerated cellulose, 0.22 μm) used in the purification of human plasma were purchased from ISOLAB. In the preparation of all aqueous solutions, 18.2 MΩ·cm deionized, ultrapure water supplied from ELGA, Purelab Option-Q lab water purification system was used.

### 2.2 Synthesis of Sialic Acid-Stabilized Gold Nanoparticles

Synthesis of sialic acid-functionalized gold nanoparticles (SA-AuNPs) was achieved by using the reducing ability of sialic acid, and the procedure proposed by Lee and co-workers was used for synthesis.^58^ A 600 μL aliquot of 0.035 M HAuCl_4_ and 500 μL of 1.0 M NaOH solutions were added to the aqueous solution of 100.0 mL of 1.0 mM sialic acid solution, and finally, the mixture was heated to 80 °C for 15 min under continuous stirring. The solution was then allowed to cool to room temperature and then the obtained gold nanoparticle solution was purified via repeated centrifugation at 9000 rpm for 20 min followed by redispersion of the pellet in 30.0 mL of deionized water and stored in a refrigerator at 4 °C for further use. In the optimization studies, different concentrations of sialic acid (0.5, 1.0, 1.5, and 2.5 mM) were used.

### 2.3 Characterization of Sialic Acid-Stabilized Gold Nanoparticles

To verify the reduction of gold ions, the reaction mixture was scanned in the range of 400–750 nm with a T80+ Double Beam UV–vis spectrophotometer (PG Instruments Ltd.). FTIR spectra were measured by preparing KBr pellets of the samples using a Bruker Alpha T FTIR spectrometer with a resolution of 4 cm^–1^. For the determination of the dispersity and shape of gold nanoparticles, a drop of sample solution was dried on the silicon wafer at room temperature, and the scanning electron microscopy (SEM) image was taken using a QUANTA 400F field emission scanning electron microscope having a resolution of 1.2 nm; the elemental composition of the nanoparticles was determined by energy-dispersive X-ray (EDX) spectrometry in METU Central Laboratory. Transmission electron microscopy (TEM) images were taken using a FEI/Tecnai G2 Spirit Biotwin 120 kV TEM (CTEM) having a resolution of 0.34 nm in METU Central Laboratory. The sizes of the nanoparticles were measured by a Malvern Mastersizer 2000 instrument in METU Central Laboratory.

### 2.4 pH Studies

The effect of the medium pH on the binding of serotonin to sialic acid was optimized. The pH of the SA-AuNP solution was about 6.7. Foremost, SA-AuNP and serotonin solutions were brought to pH values of 2.6, 3.0, 7.0, and 11.0 using 0.1 M HCl or 0.1 M NaOH solutions. UV–vis absorption spectra of nanoparticle solutions with pH values of 2.6, 3.0, 7.0, and 11.0 were monitored for 3 h in half an hour period. Two different serotonin concentrations were used for evaluating the effect of pH on the sensitivity of the measurement: 1.0 and 300.0 μM. Stock serotonin solutions having 2.0 and 600.0 μM concentrations were prepared in deionized water followed by the adjustment of the pH to 3.0, 7.0, and 11.0. 500 μL portions of pH-adjusted 2.0 and 600.0 μM serotonin solutions were added into 500 μL portions of SA-AuNP solutions at the same pH values. The absorbance spectra of the solutions having pH values of 3.0, 7.0, and 11.0 were measured 5 min after the addition of each serotonin solution. After the optimum pH of 3.0 was selected, solutions were prepared in pH 3.0 phosphate buffer (10 mM).

### 2.5 Colorimetric Determination of Serotonin by Sialic Acid-Stabilized Gold Nanoparticles

A stock serotonin solution having a 2.0 μM concentration was prepared in pH 3.0 phosphate buffer (10 mM). This stock solution was diluted proportionally to prepare serotonin solutions having a concentration between 0.05 and 1.0 μM in a pH 3.0 phosphate buffer. Into 1.0 mL of SA-AuNP solution, *x* mL of stock serotonin solutions diluted with (1 – *x*) mL of pH 3.0 phosphate buffer (10 mM) was added to obtain the desired concentration of serotonin solutions. The mixtures of SA-AuNP and serotonin solutions were incubated at various temperatures and time intervals to observe the color development of solutions due to the aggregation of nanoparticles in the presence of serotonin. The absorbance values of the resulting solutions were measured by a T80+ Double Beam UV–vis Instrument (PG Instruments Ltd.) in the range of 450–700 nm.

### 2.6 Selectivity Studies

To test the selectivity of SA-AuNPs to serotonin, stock solutions of serotonin, l-tryptophan, dopamine, galactose, l-tyrosine, glucosamine, epinephrine, oxalic acid, and mannose were prepared in pH 3.0 phosphate buffer. A final concentration of 1.0 μM serotonin, l-tryptophan, dopamine, epinephrine, and 10.0 μM galactose, l-tyrosine, glucosamine, oxalic acid, and mannose solutions in 1.0 mL portions of SA-AuNPs were incubated at room temperature for 5 min. The UV–vis absorption spectra of these solutions were measured in the range of 450–700 nm.

### 2.7 Colorimetric Determination of Serotonin in BSA Matrix

The assassination of the developed colorimetric method on use in real sample analysis was performed using bovine serum albumin (BSA) as a surrogate matrix. BSA solution was prepared by adding 4.0 g of BSA to 100 mL of PBS.^19^ The solution of BSA at pH 7.4 PBS was spiked with the standard serotonin solution. It was incubated for 1 h at pH 4.3 and 60 °C for protein precipitation, and the precipitate was treated with cold MeOH to recover the remaining serotonin from the matrix. The resulting solution was centrifuged, and the supernatant solution was collected. This procedure was repeated three times on the precipitate. The collected supernatant solutions from the spiked surrogate matrix were mixed; then, the mixture was directly added to 0.5 mL of SA-AuNP solution. The reaction temperature was kept at room temperature, and the UV–vis spectrum was taken after 5 min.

### 2.8 Colorimetric Determination of Serotonin in Biological Fluids

Human plasma aliquots were eluted from a column prepared from ZrO_2_/SiO_2_ as described in the study by Song and co-workers.^59^ For the preparation of the column material, 3.0 g of porous silica spheres (pore size 150 Å, 100–200 mesh) was treated with 30.0 mL of 0.1 M NaOH solution for 30 min and washed with deionized water. After the washing step, the silica spheres were vacuum-dried. On another setup, 10.5 g of zirconyl nitrate hydrate was rapidly added to 30.0 mL of 0.1 M HCl under vigorous stirring for 20 min. Then, the dried silica spheres were rapidly added to this solution and incubated at 70 °C for 2 h under vigorous stirring. After the incubation, the resulting particles were centrifuged three times with both deionized water and ethanol at 2000 rpm for 5 min, followed by vacuum drying at 60 °C overnight. Finally, the dried particles were put into the furnace for calcination at a heating rate of 2 °C/min from room temperature to 550 °C and kept at 550 °C for 6 h to accomplish the complete dehydration of Zr–OH to ZrO_2_ and enhancement of ZrO_2_ networks. A 5.0 mL syringe was used to build the column. 5.0 g of the synthesized ZrO_2_/SiO_2_ stationary phase was filled into the syringe, and a regenerated 0.22 μm pore size cellulose syringe filter was placed at the end of the column.

The treatment of the human aliquot was performed based on the following procedure: 300 μL of plasma aliquot spiked with 1.8 mM serotonin was injected into the column, and the elution was accomplished by fresh addition of acetonitrile in 700, 1000, and 1000 μL portions, respectively, to purify the samples from proteins and phospholipids, and the experimental setup is shown in Figure S1. The eluates were subjected to 100-fold dilution with deionized water. The appropriate amount of dilute sample solutions was directly added to 0.5 mL of SA-AuNP solution to have a final spiked amount of serotonin as 0.15, 0.50, and 0.80 μM. The absorbance values of the resulting solutions were measured by a T80+ Double Beam UV–vis Instrument (PG Instruments Ltd.) in the range of 450–700 nm. The reaction temperature was kept at room temperature, and the UV–vis absorption spectra were taken 5 min after the addition of dilute sample solution.

## 3 Results and Discussion

### 3.1 Synthesis and Characterization of Sialic Acid-Stabilized Gold Nanoparticles

Utilizing the protocol by Lee et al.,^58^ we prepared sialic acid-functionalized gold nanoparticles (SA-AuNPs) in a single reaction step. Sialic acid was employed for both reduction and stabilization during the synthesis. To ascertain the best ratio for SA-AuNPs production, we tested various sialic acid concentrations (0.5 1.0, 1.5, and 2.5 mM), while the initial Au^3+^ amount was fixed at 21 μmol. The molar ratio that produced wine red SA-AuNPs with an absorbance at 520 nm, indicating the optimal size for colorimetric analysis, was found with 1.0 mM sialic acid. Higher or lower sialic acid concentrations led to the formation of larger nanoparticles, which are not ideal for such studies. Consequently, we standardized the initial sialic acid concentration at 1.0 mM. The UV–vis absorption spectrum of these nanoparticles, which exhibit a red wine color, is presented in Figure S2, with an absorption peak around 520 nm. The optimal molar ratio of Au^3+^ to sialic acid was determined to be 0.21:1.00 at this sialic acid level.

**Figure 1:**
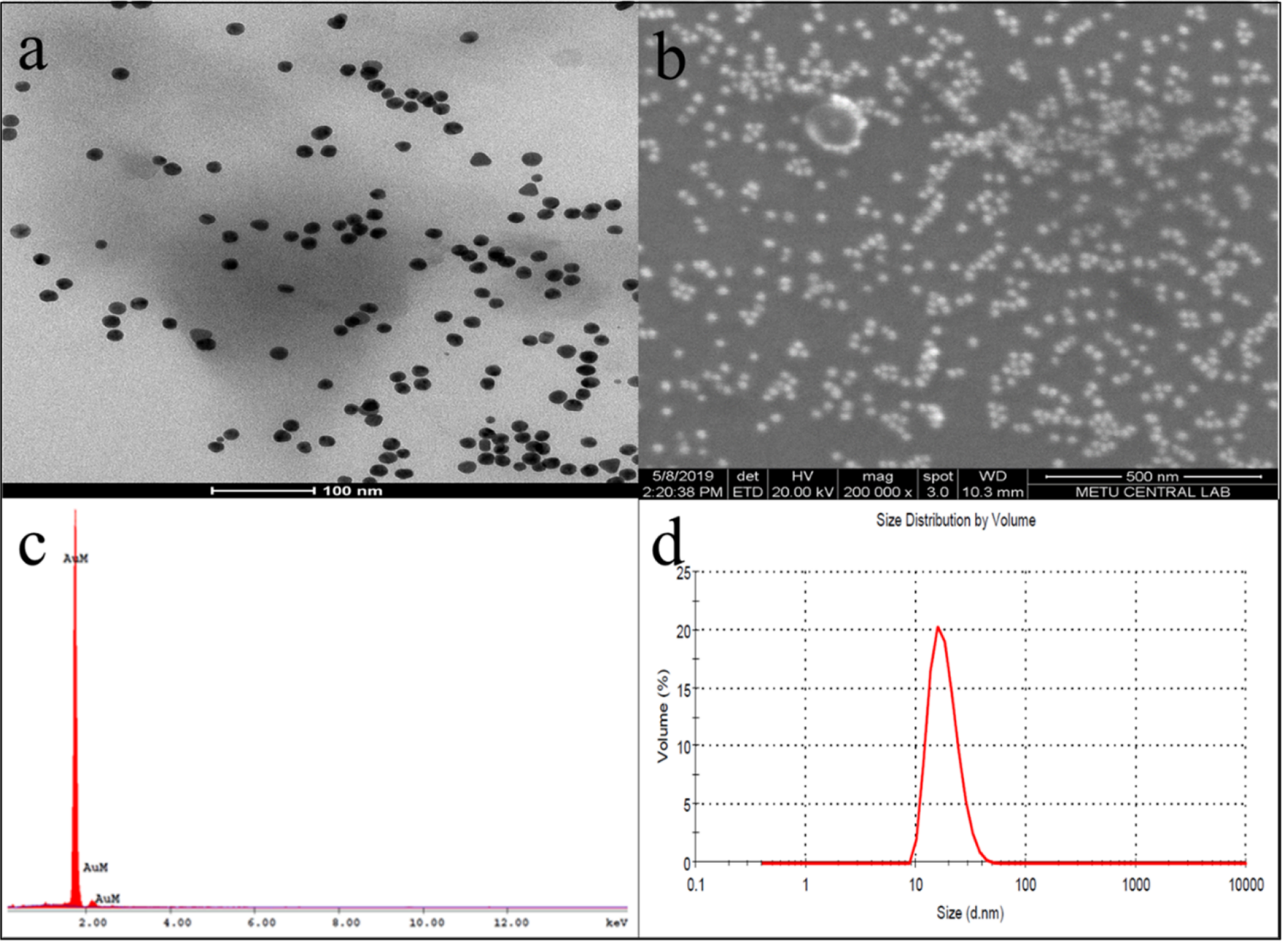
(a) TEM image, (b) SEM image, (c) EDX pattern, and (d) hydrodynamic size distribution graph of SA-AuNPs.

Figure 1a shows the transmission electron microscope (TEM) image, and Figure 1b shows the scanning electron microscope (SEM) image of SA-AuNPs. The TEM and SEM images of SA-AuNPs show the sphere-like shape of the prepared NPs. From the images, monodispersity and the spherical shape of SA-AuNPs can be seen. The strong gold signal in the energy-dispersive X-ray (EDX) pattern, Figure 1c, shows that the nanoparticles are composed of gold atoms. Additionally, Figure 1d shows that the hydrodynamic size of the SA-AuNPs was measured with a laser diffraction particle size analyzer (DLS). The average size of SA-AuNPs (diameter) was measured as 21 ± 3 nm.

**Figure 2:**
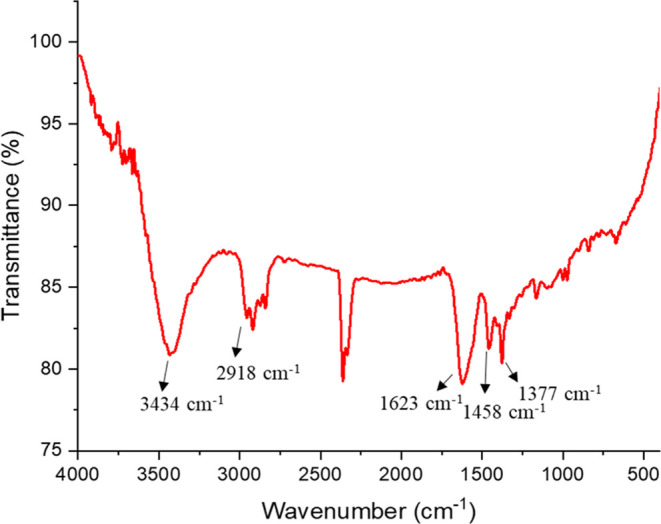
FT-IR spectrum of SA-AuNPs, and the scan number was 24.

FT-IR spectra of both sialic acid (SA) (see Figure S3) and SA-AuNPs were taken in the range of 400–4000 cm^–1^ to support the presence of sialic acid on the surface of gold nanoparticles. Sialic acid has five hydroxyl groups, one *N*-acetyl group, and one carboxyl group. The sialic acid-stabilized AuNPs yielded vibrational bands similar to the spectrum of sialic acid at 3434, 2918, 1623, 1458, and 1377 cm^–1^ as seen in Figure 2, corresponding to the O–H stretching, C–H stretching, N–H bending, C–H bending, and O–H bending bands, respectively.^60,61^ These characteristic IR bands of sialic acid observed in the FT-IR spectra of both SA and SA-AuNPs indicated that the surface of the particles was coated with sialic acid.

### 3.2 Optimization of Experimental Parameters

The performance of the colorimetric sensors is strongly influenced by the pH, reaction temperature of the medium, and duration of the measurement. All of these parameters were optimized one at a time in this study.

#### 3.2.1 Effect of pH

**Figure 3:**
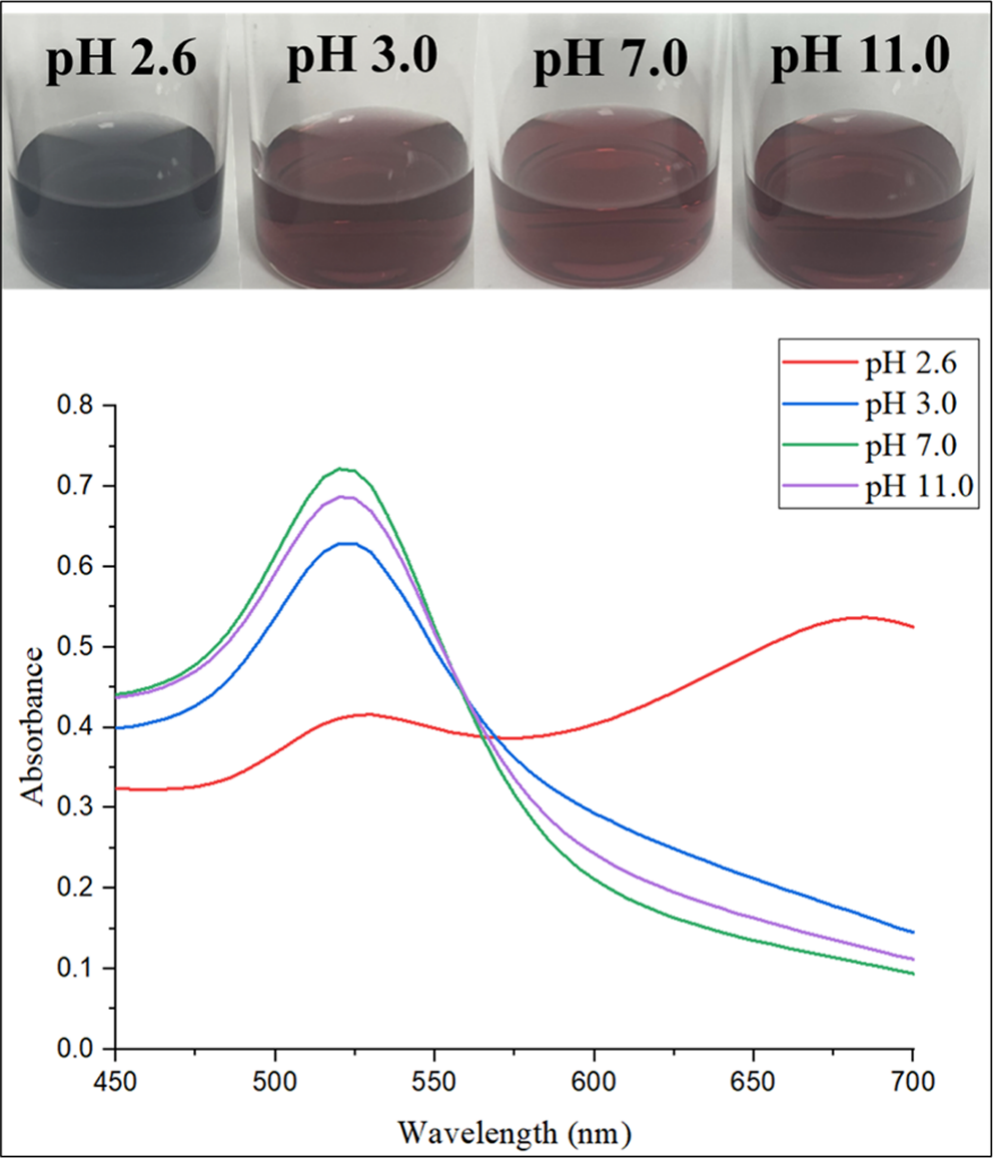
Color scheme and the absorption spectra of the SA-AuNP solutions having pH values of 2.6, 3.0, 7.0, and 11.0.

pH is important both for the noncovalent interactions and for the stability of the nanoparticle solutions. The p*K*_a_ values of the functional groups of serotonin and sialic acid were used to select the pH of the reaction medium, which, in turn, affects the ionic interactions. The selection of reaction medium pH values was done by considering the p*K*_a_ values of the functional groups of both serotonin and sialic acid. Serotonin is in cationic form below pH 9.97, while above this value it can have neutral and anionic forms.^62^ Sialic acid is neutral below pH 2.6 and negatively charged at higher pH values.^63^ pH values of 2.6, 3.0, 7.0, and 11.0 were selected considering the p*K*_a_ values of these molecules. The color scheme and absorption spectra of SA-AuNP solutions with these pH values are listed in Figure 3.

As shown in Figure 3, the most intense aggregation of SA-AuNPs was observed at pH 2.6. This is evident from the blue color of the solution at pH 2.6, while the solutions at pH 3.0, 7.0, and 11.0 remained in their original color. Our AuNPs were stabilized with sialic acid, which has a point of zero charge (PZC) of 2.6. At pH 2.6, the carboxyl groups of the sialic acid molecules on the surface of the SA-AuNPs are neutralized, resulting in zero electrostatic repulsion between the particles. This allowed the particles to aggregate rapidly. It was reported by Xiong and co-workers that the rate of change of aggregation due to pH shows a Gaussian distribution around the PZC value.^64^ This means that the rate of aggregation is highest at the PZC value and decreases as the pH deviates from the PZC value. In our case, the rate of aggregation of SA-AuNPs is highest at pH 2.6, which is the PZC value of sialic acid. Even at pH values slightly above the PZC value, the negative surface charges and repulsive forces of the AuNPs increase, which slows the aggregation rate. The UV–vis absorption spectra of SA-AuNPs solutions at pH 3.0, 7.0, and 11.0 were measured after 3 h. The absence of a second peak in the spectra of the SA-AuNPs solutions indicates that the solutions were stable at the given pH values for at least 3 h.

**Figure 4:**
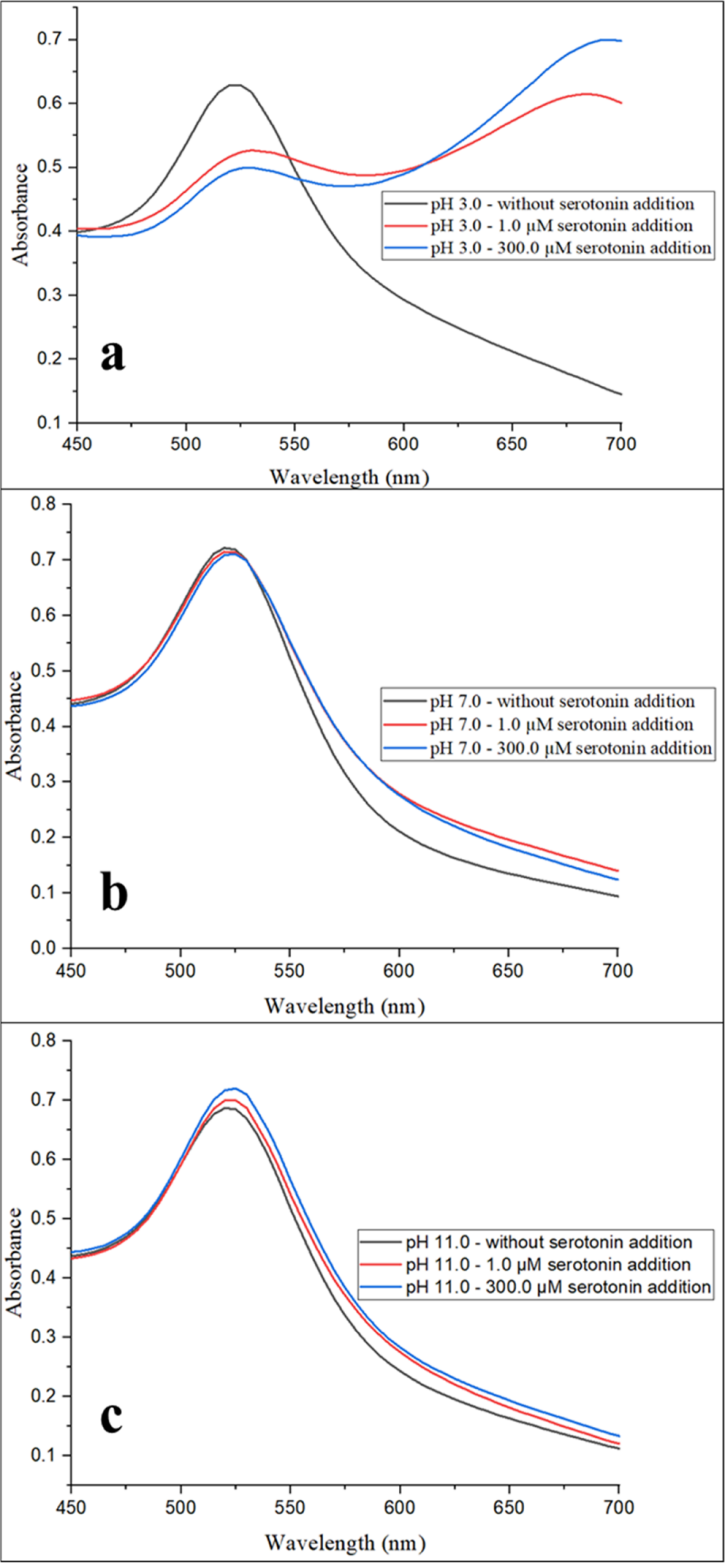
Absorption spectra of SA-AuNP solutions without and with 1.0 and 300.0 μM serotonin concentrations at pH values of (a) 3.0, (b) 7.0, and (c) 11.0.

To investigate the effect of pH on the sensitivity of colorimetric measurements, the aggregation behavior of SA-AuNP solutions was studied in the presence of 1.0 and 300.0 μM serotonin concentrations at pH values of 3.0, 7.0, and 11.0. The absorption spectra of the solutions were collected 5 min after the addition of each serotonin solution to the SA-AuNP solutions at these pH values. The results are shown in Figure 4.

Figure 4 shows that among SA-AuNP solutions at different pH values, at the same serotonin concentration, only the solution at pH 3.0 turned blue in 5 min. This color change is the result of the rapid aggregation of SA-AuNPs. This aggregation requires increased interaction between the sialic acid groups on the surface of the SA-AuNPs and the serotonin molecules. Although noncovalent bonds weaken at low pH, the enhanced aggregation can be explained by the change in the distance between the SA-AuNPs depending on the pH of the medium. At pH 7.0 and 11.0, the strong electrostatic repulsion between the SA-AuNPs keeps them apart, which increases the interaction volume between them.^65^ This reduces the chance of serotonin molecules bridging the SA-AuNPs through their sialic acid groups, which is necessary for aggregation. At pH 3.0, the electrostatic repulsion between SA-AuNPs is weaker. This allows the SA-AuNPs to come closer together, which decreases the interaction volume between them (see Figure S4). As a result, the serotonin molecules can bind to the sialic acid groups on adjacent SA-AuNPs more easily, which causes the AuNPs to aggregate rapidly.

As shown in Figure 4a, at pH 3.0, a second peak was observed in the spectra of the SA-AuNPs due to aggregation of the NPs, even in the presence of a low serotonin concentration of 1.0 μM. In contrast, at pH 7.0 (Figure 4b) and 11.0 (Figure 4c), no second peak formation was observed in their spectra even when the serotonin concentration was increased by 300 times. Therefore, to enhance the sensitivity of the measurements, pH 3.0 was chosen as the pH for this study.

#### 3.2.2 Temperature Dependence

**Figure 5:**
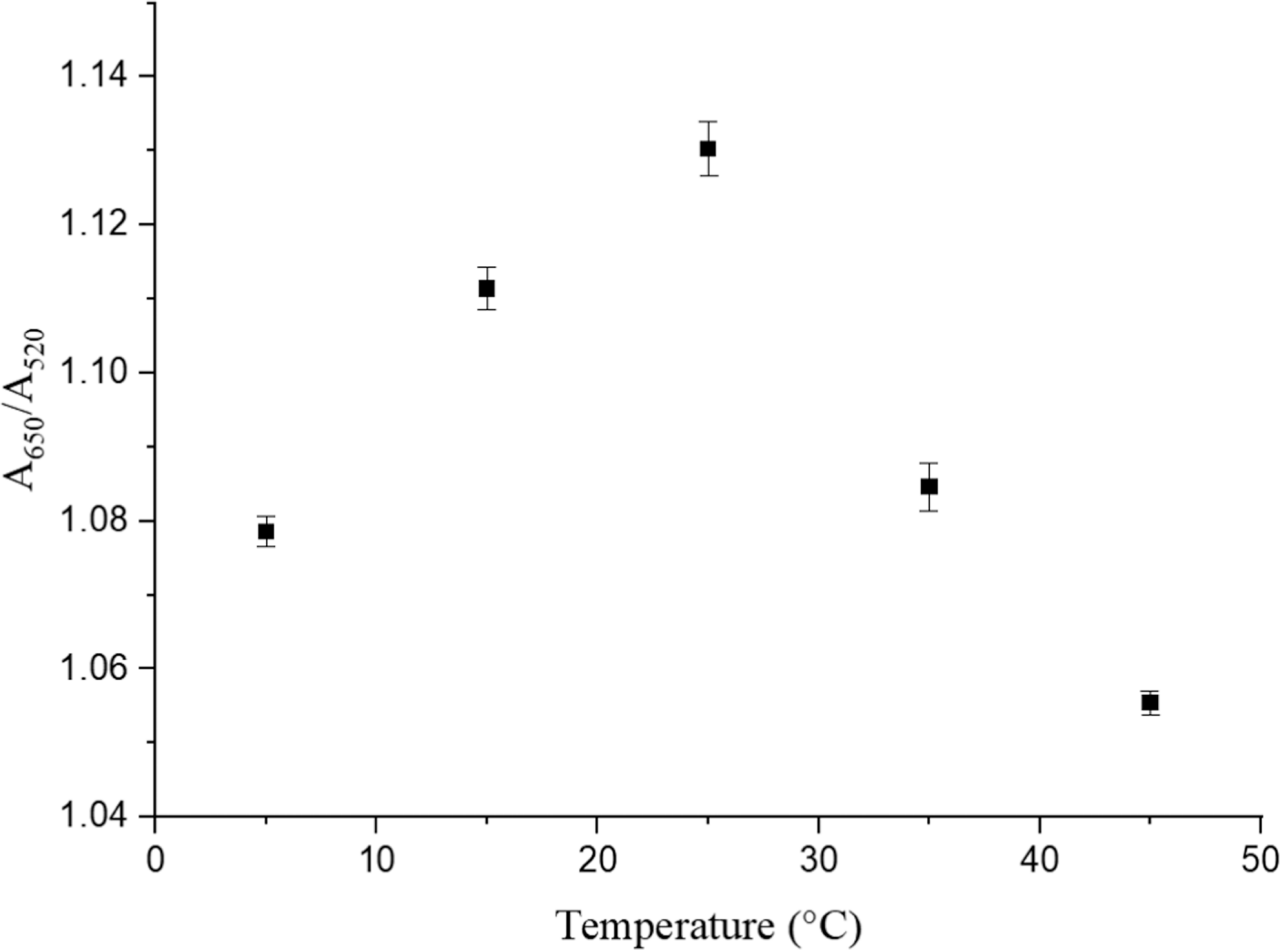
Absorbance ratio (*A*_650_/*A*_520_) was measured at different temperatures after the addition of 1.0 μM serotonin at pH 3.0, and the standard deviation was based on three measurements.

The role of temperature is significant in chemical reactions based on hydrogen bonding interactions. The absorbance ratio of *A*_650_/*A*_520_ was measured at various temperatures 5 min after the addition of 1.0 μM serotonin to the SA-AuNP solution at pH 3.0 as shown in Figure 5. The rapid increase of *A*_650_/*A*_520_ reached a maximum at 25 °C followed by a sharp decrease at temperatures higher than 25 °C. Therefore, the temperature was kept constant at 25 °C throughout this study.

#### 3.2.3 Time Dependence

**Figure 6:**
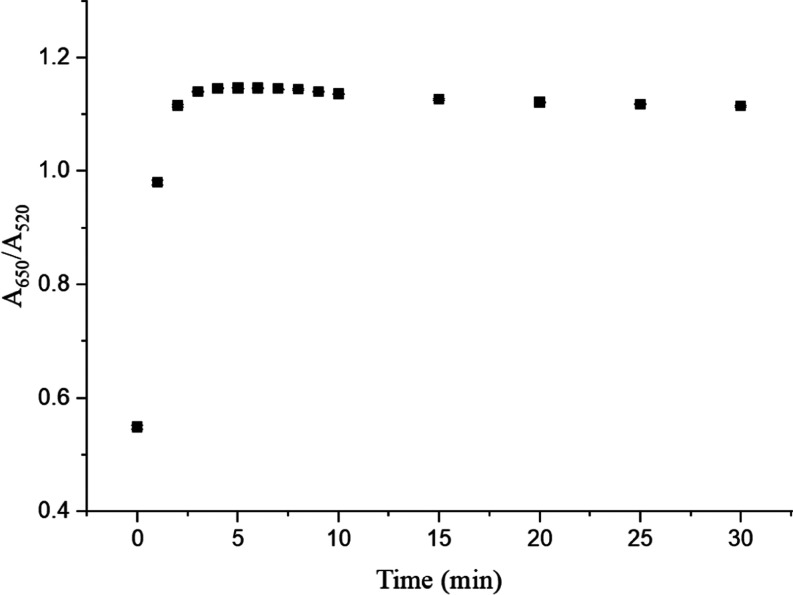
Time dependence of the absorbance ratio (*A*_650_/*A*_520_), the standard deviation was based on three measurements.

The incubation time is another parameter that needs to be optimized. The absorbance ratio of *A*_650_/*A*_520_ after the addition of 1.0 μM serotonin to the SA-AuNPs solution at pH 3.0 was measured in 5 min increments. The value of the ratio reached its maximum value in the first 5 min, and then the ratio did not change drastically as shown in Figure 6. Therefore, the optimum incubation time was chosen as 5 min to complete the analysis as fast as possible with the highest signal.

### 3.3 Determination of Serotonin by Using Sialic Acid-Stabilized Gold Nanoparticles

**Figure 7:**
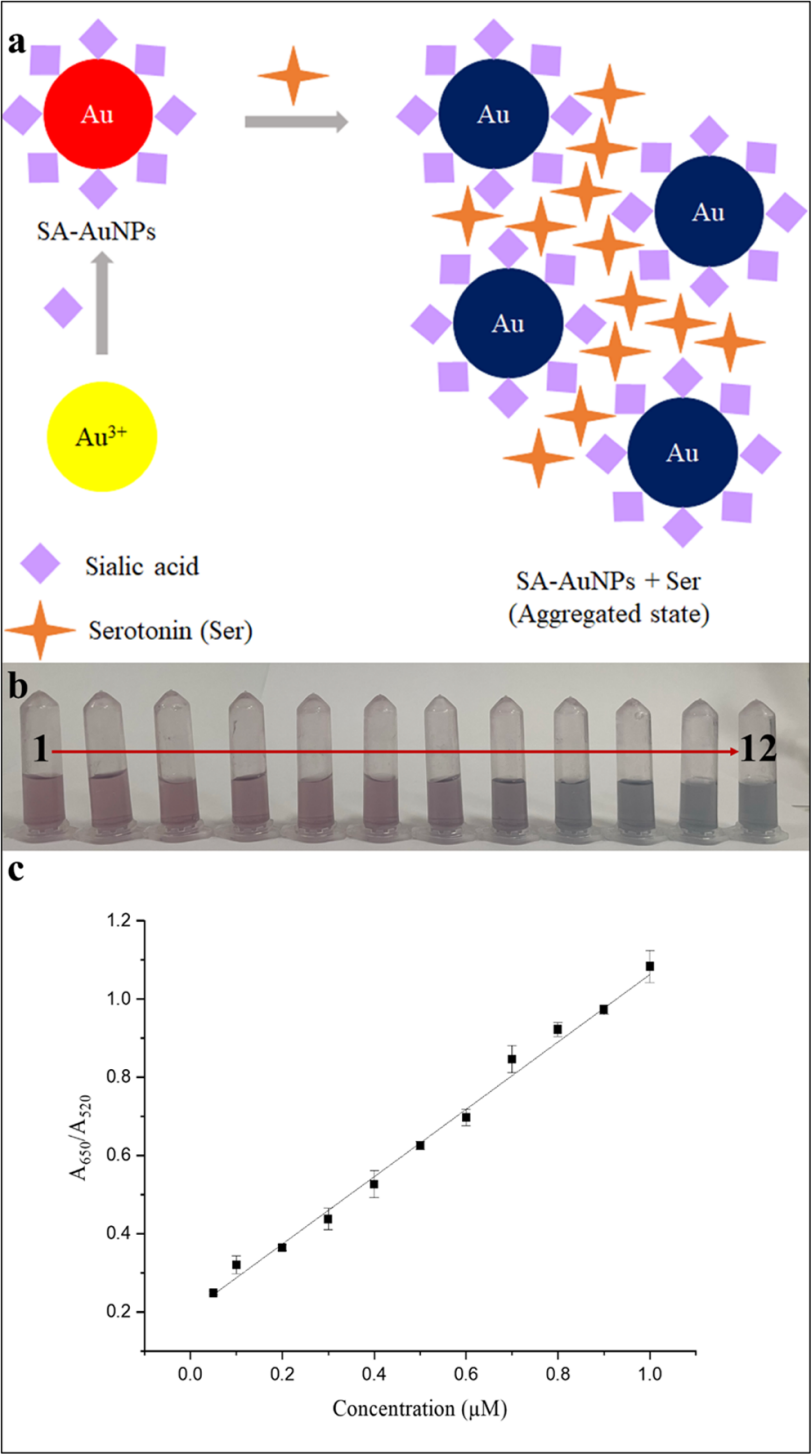
(a) Scheme for serotonin and SA-AuNPs interaction; (b) visual color change of SA-AuNP solutions after addition of serotonin standard solutions (2–12) and color of blank solution (1); (c) calibration plot of serotonin standard solutions (2–12) between 0.05 and 1.0 μM.

After optimizing the experimental conditions, we investigated the effect of serotonin concentration on the absorption spectra of SA-AuNPs within the range of 0.05–1.0 μM serotonin. We expected a red shift in the spectrum as larger aggregates were formed due to the noncovalent complex formation between sialic acid and serotonin. To quantify the amount of serotonin, we took the ratio of absorbance at 650 nm to absorbance at 520 nm as the ordinate of the calibration graph. The reaction cartoon of serotonin with SA-AuNPs and the visual change of color of SA-AuNPs solutions following the addition of serotonin having various concentrations at 25 °C are shown in Figure 7a,b, respectively. The calibration plot is given in Figure 7c. Each absorbance measurement was performed 5 min after the addition of a serotonin solution.

The absorbance of each solution was measured three times, and the standard deviation of the signal was shown for each solution in the calibration plot. The slope of the calibration line at the specified concentration range was found as 0.861 ± 0.014 μM^–1^, the intercept as 0.201 unitless, and *R*^2^ as 0.998.

A blank solution was prepared by dispersing SA-AuNPs in a pH 3.0 buffer solution. Based on the 3*s* and 10*s* criteria, the limit of detection limit was calculated as 0.02 μM, and the limit of quantification was 0.06 μM, where *s* is the standard deviation of 10 replicates of *A*_650_/*A*_520_ ratio from the blank measurements.

Compared to the determination studies given in Section 1, the proposed method can detect much lower concentrations of serotonin than other plasmonic (15-fold^45^ and 5-fold^44^ lower), colorimetric (115-fold^46^ lower), and even some fluorimetric,^27,32^ electrochemical,^25^ and mass spectrometric^19^ methods.

Table 1 shows the comparison of various methods for the determination of serotonin with the proposed method. A plasmonic study used the aptamer-gold nanoparticle conjugates prepared by physical adsorption of the DNA on the nanoparticle’s surface as a sensor for serotonin.^45^ In another plasmonic study, a bifunctionalized probe was designed by functionalizing gold nanoparticles with dithiobis(succinimidyl propionate) (DSP) and *N*-acetyl-l-cysteine (NALC).^44^ In a colorimetric study, the colored product from the reaction of serotonin and Ehrlich’s reagent was measured by spectrophotometry.^46^ A study used a cage-based metal–organic framework (NKU-67-Eu) as an artificial chemical receptor to recognize serotonin due to its well-matched energy levels with serotonin.^27^ Lanthanide-doped metal–organic frameworks were also suggested as a ratiometric fluorescence biosensor for visual and ultrasensitive detection of serotonin.^32^ In an electrochemical study, the boron/nitrogen codoped with diamond graphene nanowalls (DGNW) integrated with the screen-printed graphene electrode (SPGE) was designed as a model system for the detection of serotonin.^25^ Lastly, the liquid chromatography-tandem mass spectrometry (LC-MS/MS) method was reported as a report quick, accurate, and reliable method for the quantitative determination of serotonin.^19^ From Table 1, it can be concluded that the detection limit of the proposed method is much lower than that of the others, revealing the advantage of this method.

**Table 1:** Various Methods for Determination of Serotonin

method	limit of detection (LOD), μM	linear range	reference
plasmonic via SA-AuNPs	0.02	0.05–1.0 μM	this work
plasmonic Apt-AuNPs	0.300	750 nM to 2.5 μM	(45)
plasmonic bifunctionalized AuNPs	0.1	0–1 μM	(44)
colorimetric with Ehrlich’s reagent	2.3	0.025–0.5 mM	(46)
fluorimetric (cage-based metal–organic framework)	0.036	0.5–1.4 μM, >1.9 μM	(27)
fluorimetric	0.57	0–200 μM	(32)
electrochemical	0.28	1–500 μM	(25)
mass spectrometric		0.57–14.2 μM	(19)

### 3.4 Selectivity Studies

**Figure 8:**
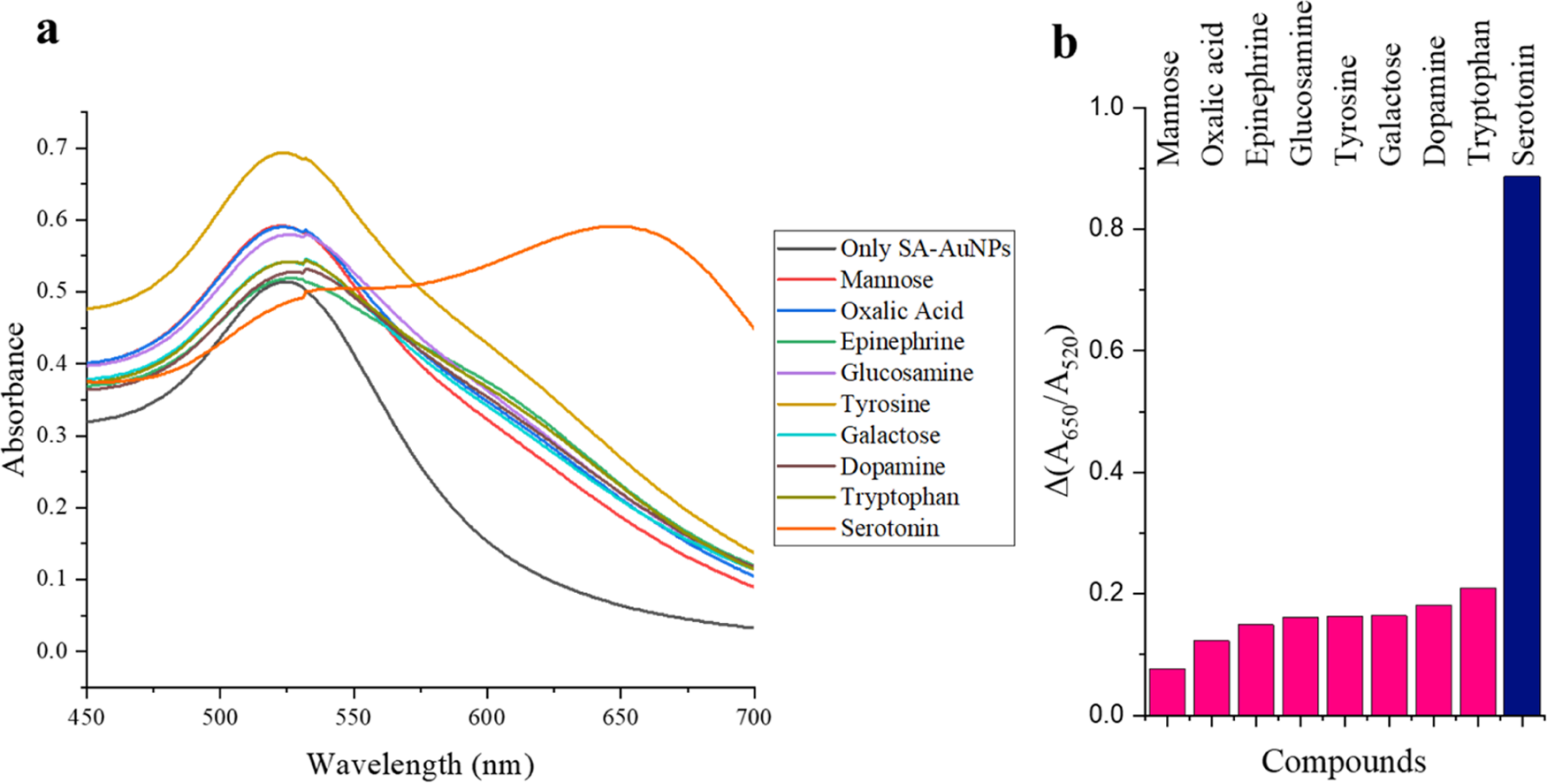
(a) Absorption spectra of SA-AuNPs and (b) the difference between the absorbance ratio of SA-AuNPs at 650–520 nm before and 5 min after the addition of serotonin and interfering species to the SA-AuNP solution.

A selectivity study was performed with a range of potential interfering species such as l-tryptophan (precursor to serotonin), dopamine and epinephrine (neurotransmitters as serotonin), l-tyrosine, glucosamine, galactose, mannose, and oxalic acid. The presence of serotonin resulted in a significant color change (blue), while the addition of potential interferents had no visible effect on the color of the SA-AuNP solution. The absorption spectra of SA-AuNP solutions can be seen in Figure 8a. The difference between the absorbance ratio of SA-AuNPs at 650–520 nm before and 5 min after the addition of serotonin and interfering species to the SA-AuNPs solution is displayed in Figure 8b. In the preparation of the bar graph in Figure 8b, the potential influence of tartrate ions in the interference study of epinephrine was excluded. This was done by using the difference in absorbance ratio at 650–520 nm between the SA-AuNPs solution after the addition of epinephrine-bitartrate and sodium bitartrate solutions.

In Figure 8a, it can be observed that the introduction of serotonin into the SA-AuNP solutions led to a noticeable aggregation of the nanoparticles. This is indicated by the appearance of a second peak at 650 nm and a significant increase in the absorbance ratio (*A*_650_/*A*_520_). However, no such aggregation occurred when other interfering species were added to the SA-AuNP solutions. The interfering species including dopamine and epinephrine, known for their competitive nature in interfering with serotonin, did not cause a significant intervention in the SA-AuNP solution spectrum. This indicates that the method is selective for serotonin. Moreover, the specificity of the colorimetric method for serotonin is evident, as l-tryptophan, which is structurally very similar, does not notably interfere. Therefore, the proposed method for determining serotonin has shown promising results in selectivity and specificity. As mentioned earlier, it has been suggested that sialic acid possesses a unique binding capacity for serotonin. The presence of the *N*-acetate group, C-7–C-9 chain, and carboxyl group in the molecular structure of sialic acid is believed to be necessary for the sialic acid–serotonin interaction. It is thought that the formation of multiple hydrogen bonds between sialic acid and serotonin may explain the strength and specificity of this interaction.

### 3.5 Colorimetric Determination of Serotonin in the BSA Matrix

The developed colorimetric method was applied to a surrogate matrix, bovine serum albumin (BSA). The recoveries are within the range of 96.2–104.8% (see Table S1) showing that the developed method can be applied to biological fluids such as human plasma. As a consequence, the study was enriched by applying the method to human plasma.

### 3.6 Application of the Colorimetric Method to Human Plasma

We applied the method proposed in this study to quantify the serotonin levels in spiked human plasma aliquots. When we analyzed the plasma aliquots directly, with or without diluting them with water, we did not observe a red shift in the absorption spectrum due to the plasmon coupling of aggregated SA-AuNPs. This was probably due to the absence of the nonspecific adsorption of proteins onto the SA-AuNPs. Protein precipitation with organic solvents is a common sample cleanup technique in bioanalysis.^66^ We tried using acetonitrile as an organic solvent to precipitate the proteins, but even though we obtained a clear supernatant after centrifugation, the recovery of the plasma aliquots was still low. To eliminate proteins and other endogenous cellular components such as phospholipids from the plasma, we prepared a ZrO_2_/SiO_2_ column.^59,67^ The serotonin-spiked plasma aliquots were eluted from the column with acetonitrile. ZrO_2_ exhibits anion exchange properties toward polyoxy anions such as phosphates, sulfates, and carboxylates.^68^ The Zr atoms in the column act as Lewis acids and interact with the phosphate groups of the phospholipids, which are Lewis bases. This interaction allows the phospholipids to be retained on the column, while the coagulated proteins are physically entrapped. Other molecules are eluted with acetonitrile. After collecting the samples from the column, we calculated the recoveries using the developed method. Table 2 shows the recovery results for human plasma. The recoveries were within the range of 90.5–104.2%, which shows that the developed method has promising feasibility for the rapid and selective determination of serotonin in biological fluids.

**Table 2:** Results for the Determination of Serotonin in the Human Plasma

	spiked amount (μM)	found amount (μM)^a^	recovery (%)^a^
treated human plasma	0.15	0.156 ± 0.018	104.2 ± 11.7
	0.50	0.453 ± 0.039	90.5 ± 7.8
	0.80	0.752 ± 0.064	94.0 ± 8.0

a Mean ± standard deviation, *n* = 3.

## 4 Conclusions

We have developed a new colorimetric method that uses sialic-acid-coated gold nanoparticles (SA-AuNPs) with plasmonic properties for the determination of serotonin. The method works by detecting the color change that occurs in SA-AuNP solutions when they aggregate in the presence of serotonin. This aggregation causes a red shift in the absorption spectrum of the SA-AuNPs, which can be measured with a UV–vis spectrometer. We also tested the selectivity of the method to serotonin by testing it with various other biomolecules such as l-tryptophan, dopamine, galactose, l-tyrosine, glucosamine, epinephrine, oxalic acid, and mannose. The results demonstrated high selectivity for serotonin, as there was no significant color change observed in the presence of other interferants. The method has been successfully applied for determining the serotonin levels in spiked processed human plasma, with recoveries ranging from 90.5 to 104.2%. Therefore, the proposed colorimetric SA-AuNP method is a cost-effective, simple, fast, reliable, sensitive, and selective alternative for the determination of serotonin in clinical applications.

## Supporting Information Available

Setup for ZrO_2_/SiO_2_ column, Figure S1; UV–vis absorption spectrum of SA-AuNPs solution, Figure S2; FT-IR spectrum of solid SA, Figure S3; the effect of pH on the interaction volume of nanoparticles, Figure S4; colorimetric determination of serotonin in the BSA matrix, Table S1 (PDF).

## References

[ref1] Jones L. A.; Sun E. W.; Martin A. M.; Keating D. J. The Ever-Changing Roles of Serotonin. Int. J. Biochem. Cell Biol. 2020, 125, 105776 10.1016/j.biocel.2020.105776. 32479926

[ref2] Karayol R.; Medrihan L.; Warner-Schmidt J. L.; Fait B. W.; Rao M. N.; Holzner E. B.; Greengard P.; Heintz N.; Schmidt E. F. Serotonin Receptor 4 in the Hippocampus Modulates Mood and Anxiety. Mol. Psychiatry 2021, 26 (6), 2334–2349. 10.1038/s41380-020-00994-y. 33441982 PMC8275670

[ref3] Vaseghi S.; Arjmandi-Rad S.; Nasehi M.; Zarrindast M. R. Cannabinoids and Sleep-Wake Cycle: The Potential Role of Serotonin. Behav. Brain Res. 2021, 412, 113440 10.1016/j.bbr.2021.113440. 34216647

[ref4] Nonogaki K. The Regulatory Role of the Central and Peripheral Serotonin Network on Feeding Signals in Metabolic Diseases. Int. J. Mol. Sci. 2022, 23 (3), 1600 10.3390/ijms23031600. 35163521 PMC8836087

[ref5] Lacivita E.; Niso M.; Mastromarino M.; Garcia Silva A.; Resch C.; Zeug A.; Loza M. I.; Castro M.; Ponimaskin E.; Leopoldo M. Knowledge-Based Design of Long-Chain Arylpiperazine Derivatives Targeting Multiple Serotonin Receptors as Potential Candidates for Treatment of Autism Spectrum Disorder. ACS Chem. Neurosci. 2021, 12 (8), 1313–1327. 10.1021/acschemneuro.0c00647. 33792287

[ref6] Fanelli D.; Weller G.; Liu H. New Serotonin-Norepinephrine Reuptake Inhibitors and Their Anesthetic and Analgesic Considerations. Neurol. Int. 2021, 13 (4), 497–509. 10.3390/neurolint13040049. 34698218 PMC8544373

[ref7] Atmaca M. Selective Serotonin Reuptake Inhibitor-Induced Sexual Dysfunction: Current Management Perspectives. Neuropsychiatr. Dis. Treat. 2020, 16, 1043–1050. 10.2147/NDT.S185757. 32368066 PMC7182464

[ref8] Bahr F. S.; Ricke-Hoch M.; Ponimaskin E.; Müller F. E. Serotonin Receptors in Myocardial Infarction: Friend or Foe?. ACS Chem. Neurosci. 2024, 15, 1619 10.1021/acschemneuro.4c00031. 38573542 PMC11027101

[ref9] Atici T.; Kamaç M. B.; Yilmaz M.; Kabaca A. Y. Zinc Oxide Nanorod/Polymethylene Blue (Deep Eutectic Solvent)/Gold Nanoparticles Modified Electrode for Electrochemical Determination of Serotonin (5-HT). ElectrochimActa 2023, 458, 142484 10.1016/j.electacta.2023.142484.

[ref10] Maximino C.; Puty B.; Benzecry R.; Araújo J.; Lima M. G.; De Jesus Oliveira Batista E.; Renata De Matos Oliveira K.; Crespo-Lopez M. E.; Herculano A. M. Role of Serotonin in Zebrafish (Danio Rerio) Anxiety: Relationship with Serotonin Levels and Effect of Buspirone, WAY 100635, SB 224289, Fluoxetine and Para-Chlorophenylalanine (PCPA) in Two Behavioral Models. Neuropharmacology 2013, 71, 83–97. 10.1016/j.neuropharm.2013.03.006. 23541719

[ref11] Ren C.; Liu J.; Zhou J.; Liang H.; Wang Y.; Sun Y.; Ma B.; Yin Y. Low Levels of Serum Serotonin and Amino Acids Identified in Migraine Patients. Biochem. Biophys. Res. Commun. 2018, 496 (2), 267–273. 10.1016/j.bbrc.2017.11.203. 29294327

[ref12] Park J.-I.; Lee I. H.; Lee S. J.; Kwon R. W.; Choo E. A.; Nam H. W.; Lee J. B. Effects of Music Therapy as an Alternative Treatment on Depression in Children and Adolescents with ADHD by Activating Serotonin and Improving Stress Coping Ability. BMC Complement. Med. Ther. 2023, 23 (1), 73 10.1186/s12906-022-03832-6. 36879223 PMC9987133

[ref13] Spiller R.; Lam C. An Update on Post-Infectious Irritable Bowel Syndrome: Role of Genetics, Immune Activation, Serotonin and Altered Microbiome. J. Neurogastroenterol. Motil. 2012, 18 (3), 258–268. 10.5056/jnm.2012.18.3.258. 22837873 PMC3400813

[ref14] Spadaro A.; Scott K. R.; Koyfman A.; Long B. High Risk and Low Prevalence Diseases: Serotonin Syndrome. Am. J. Emerg. Med. 2022, 61, 90–97. 10.1016/j.ajem.2022.08.030. 36057215

[ref15] Depoilly T.; Leroux R.; Andrade D.; Nicolle R.; Dioguardi Burgio M.; Marinoni I.; Dokmak S.; Ruszniewski P.; Hentic O.; Paradis V.; De Mestier L.; Perren A.; Couvelard A.; Cros J. Immunophenotypic and Molecular Characterization of Pancreatic Neuroendocrine Tumors Producing Serotonin. Mod. Pathol. 2022, 35 (11), 1713–1722. 10.1038/s41379-022-01110-x. 35739266

[ref16] Shen Y.; Luo X.; Li H.; Chen Z.; Guan Q.; Cheng L. Simple and Reliable Serotonin Assay in Human Serum by LC-MS/MS Method Coupled with One Step Protein Precipitation for Clinical Testing in Patients with Carcinoid Tumors. J. Chromatogr. B 2020, 1158, 122395 10.1016/j.jchromb.2020.122395. 33091677

[ref17] Peterson Z. D.; Lee M. L.; Graves S. W. Determination of Serotonin and Its Precursors in Human Plasma by Capillary Electrophoresis–Electrospray Ionization–Time-of-Flight Mass Spectrometry. J. Chromatogr. B 2004, 810 (1), 101–110. 10.1016/S1570-0232(04)00597-5. 15358313

[ref18] De Jong W. H. A.; Wilkens M. H. L. I.; De Vries E. G. E.; Kema I. P. Automated Mass Spectrometric Analysis of Urinary and Plasma Serotonin. Anal. Bioanal. Chem. 2010, 396 (7), 2609–2616. 10.1007/s00216-010-3466-5. 20140664 PMC2841759

[ref19] Virág D.; Király M.; Drahos L.; Édes A. E.; Gecse K.; Bagdy G.; Juhász G.; Antal I.; Klebovich I.; Dalmadi Kiss B.; Ludányi K. Development, Validation and Application of LC–MS/MS Method for Quantification of Amino Acids, Kynurenine and Serotonin in Human Plasma. J. Pharm. Biomed. Anal. 2020, 180, 4–11. 10.1016/j.jpba.2019.113018. 31851908

[ref20] Wu K.; Fei J.; Hu S. Simultaneous Determination of Dopamine and Serotonin on a Glassy Carbon Electrode Coated with a Film of Carbon Nanotubes. Anal. Biochem. 2003, 318 (1), 100–106. 10.1016/S0003-2697(03)00174-X. 12782037

[ref21] Swamy B. E. K.; Venton B. J. Carbon Nanotube-Modified Microelectrodes for Simultaneous Detection of Dopamine and Serotonin in Vivo. Analyst 2007, 132 (9), 876–884. 10.1039/b705552h. 17710262

[ref22] Amatatongchai M.; Sitanurak J.; Sroysee W.; Sodanat S.; Chairam S.; Jarujamrus P.; Nacapricha D.; Lieberzeit P. A. Highly Sensitive and Selective Electrochemical Paper-Based Device Using a Graphite Screen-Printed Electrode Modified with Molecularly Imprinted Polymers Coated Fe3O4@Au@SiO2 for Serotonin Determination. Anal. Chim. Acta 2019, 1077, 255–265. 10.1016/j.aca.2019.05.047. 31307717

[ref23] Goyal R. N.; Gupta V. K.; Oyama M.; Bachheti N. Gold Nanoparticles Modified Indium Tin Oxide Electrode for the Simultaneous Determination of Dopamine and Serotonin: Application in Pharmaceutical Formulations and Biological Fluids. Talanta 2007, 72 (3), 976–983. 10.1016/j.talanta.2006.12.029. 19071712

[ref24] Mahato K.; Purohit B.; Bhardwaj K.; Jaiswal A.; Chandra P. Novel Electrochemical Biosensor for Serotonin Detection Based on Gold Nanorattles Decorated Reduced Graphene Oxide in Biological Fluids and in Vitro Model. Biosens. Bioelectron. 2019, 142, 111502 10.1016/j.bios.2019.111502. 31326860

[ref25] Boonkaew S.; Dettlaff A.; Bogdanowicz R.; Jönsson-Niedzió M. Electrochemical Determination of Neurotransmitter Serotonin Using Boron/Nitrogen Co-Doped Diamond-Graphene Nanowall-Structured Particles. J. Electroanal. Chem. 2022, 926, 116938 10.1016/j.jelechem.2022.116938.

[ref26] Ramon-Marquez T.; Medina-Castillo A. L.; Fernandez-Gutierrez A.; Fernandez-Sanchez J. F. A Novel Optical Biosensor for Direct and Selective Determination of Serotonin in Serum by Solid Surface-Room Temperature Phosphorescence. Biosens. Bioelectron. 2016, 82, 217–223. 10.1016/j.bios.2016.04.008. 27085954

[ref27] Min H.; Sun T.; Cui W.; Han Z.; Yao P.; Cheng P.; Shi W. Cage-Based Metal-Organic Framework as an Artificial Energy Receptor for Highly Sensitive Detection of Serotonin. Inorg. Chem. 2023, 62 (22), 8739–8745. 10.1021/acs.inorgchem.3c01025. 37224141

[ref28] Borse S.; Murthy Z. V. P.; Park T. J.; Kailasa S. K. The Influence of Surface Ligand Chemistry for the Synthesis of Blue Fluorescent Gold Nanoclusters for the Detection of Serotonin in Biofluids. New J. Chem. 2023, 47 (6), 3075–3083. 10.1039/D2NJ04797G.

[ref29] Puozzo C.; Filaquier C.; Zorza G. Determination of Milnacipran, a Serotonin and Noradrenaline Reuptake Inhibitor, in Human Plasma Using Liquid Chromatography with Spectrofluorimetric Detection. J. Chromatogr. B 2004, 806 (2), 221–228. 10.1016/j.jchromb.2004.03.063. 15171932

[ref30] Crawford N.; Rudd B. T. A Spectrophotofluorimetric Method for the Determination of Serotonin (5-Hydroxytryptamine) in Plasma. Clin. Chim. Acta 1962, 7 (1), 114–121. 10.1016/0009-8981(62)90127-4. 13882071

[ref31] De Benedetto G. E.; Fico D.; Pennetta A.; Malitesta C.; Nicolardi G.; Lofrumento D. D.; De Nuccio F.; La Pesa V. A Rapid and Simple Method for the Determination of 3,4-Dihydroxyphenylacetic Acid, Norepinephrine, Dopamine, and Serotonin in Mouse Brain Homogenate by HPLC with Fluorimetric Detection. J. Pharm. Biomed. Anal. 2014, 98, 266–270. 10.1016/j.jpba.2014.05.039. 24971521

[ref32] Song L.; Tian F.; Liu Z. Lanthanide Doped Metal-Organic Frameworks as a Ratiometric Fluorescence Biosensor for Visual and Ultrasensitive Detection of Serotonin. J. Solid State Chem. 2022, 312, 123231 10.1016/j.jssc.2022.123231.

[ref33] Wang W.; Zhang B.; Zhang Y.; Ma P.; Wang X.; Sun Y.; Song D.; Fei Q. Colorimetry and SERS Dual-Mode Sensing of Serotonin Based on Functionalized Gold Nanoparticles. Spectrochim. Acta, Part A 2021, 261, 120057 10.1016/j.saa.2021.120057. 34119772

[ref34] Do P. Q. T.; Huong V. T.; Phuong N. T. T.; Nguyen T. H.; Ta H. K. T.; Ju H.; Phan T. B.; Phung V. D.; Trinh K. T. L.; Tran N. H. T. The Highly Sensitive Determination of Serotonin by Using Gold Nanoparticles (Au NPs) with a Localized Surface Plasmon Resonance (LSPR) Absorption Wavelength in the Visible Region. RSC Adv. 2020, 10 (51), 30858–30869. 10.1039/D0RA05271J. 35516028 PMC9056339

[ref35] Chen Y.; Zhao C.; Yue G.; Yang Z.; Wang Y.; Rao H.; Zhang W.; Jin B.; Wang X. A Highly Selective Chromogenic Probe for the Detection of Nitrite in Food Samples. Food Chem. 2020, 317, 126361 10.1016/j.foodchem.2020.126361. 32070846

[ref36] Fernandes R. S.; Sanjay C.; Ghosh B.; Dey N. Sulfide-Induced Concentration-Dependent Distinct Optical Response: Unique Chromogenic Probe Developed for Analyzing Fecal Contamination in Water and Intracellular Imaging Applications. ACS Sustainable Chem. Eng. 2024, 12, 4922–4932. 10.1021/acssuschemeng.3c07719.

[ref37] Vilela D.; González M. C.; Escarpa A. Sensing Colorimetric Approaches Based on Gold and Silver Nanoparticles Aggregation: Chemical Creativity behind the Assay. A Review. Anal. Chim. Acta 2012, 751, 24–43. 10.1016/j.aca.2012.08.043. 23084049

[ref38] Cui Y.; Zhao J.; Li H. Chromogenic Mechanisms of Colorimetric Sensors Based on Gold Nanoparticles. Biosensors 2023, 13 (8), 801 10.3390/bios13080801. 37622887 PMC10452725

[ref39] Kailasa S. K.; Koduru J. R.; Desai M. L.; Park T. J.; Singhal R. K.; Basu H. Recent Progress on Surface Chemistry of Plasmonic Metal Nanoparticles for Colorimetric Assay of Drugs in Pharmaceutical and Biological Samples. TrAC, Trends Anal. Chem. 2018, 105, 106–120. 10.1016/j.trac.2018.05.004.

[ref40] Moslemi A.; Sansone L.; Esposito F.; Campopiano S.; Giordano M.; Iadicicco A. Optical Fiber Probe Based on LSPR for the Detection of Pesticide Thiram. Opt. Laser Technol. 2024, 175, 110882 10.1016/j.optlastec.2024.110882.

[ref41] Singh R.; Zhang W.; Liu X.; Zhang B.; Kumar S. WaveFlex Biosensor: MXene-Immobilized W-Shaped Fiber-Based LSPR Sensor for Highly Selective Tyramine Detection. Opt. Laser Technol. 2024, 171, 110357 10.1016/j.optlastec.2023.110357.

[ref42] Abdi G.; Bahador H. High Sensitivity and Optimum Design of LSPR-Based Sensors by Coupled Nano-Rings for Cancer Detection. Opt. Lasers Eng. 2024, 174, 107975 10.1016/j.optlaseng.2023.107975.

[ref43] Lertvachirapaiboon C.; Baba A.; Shinbo K.; Kato K. Colorimetric Detection Based on Localized Surface Plasmon Resonance for Determination of Chemicals in Urine. Anal. Sci. 2021, 37 (7), 929–940. 10.2116/analsci.20R005. 33132235

[ref44] Godoy-Reyes T. M.; Llopis-Lorente A.; Costero A. M.; Sancenón F.; Gaviña P.; Martínez-Máñez R. Selective and Sensitive Colorimetric Detection of the Neurotransmitter Serotonin Based on the Aggregation of Bifunctionalised Gold Nanoparticles. Sens. Actuators, B 2018, 258, 829–835. 10.1016/j.snb.2017.11.181.

[ref45] Chávez J.; Hagen J. A.; Kelley-Loughnane N. Fast and Selective Plasmonic Serotonin Detection with Aptamer-Gold Nanoparticle Conjugates. Sensors 2017, 17 (4), 681 10.3390/s17040681. 28346350 PMC5419794

[ref46] Jin Q.; Shan L.; Yue J.; Wang X. Spectrophotometric Determination of Total Serotonin Derivatives in the Safflower Seeds with Ehrlich’s Reagent and the Underlying Color Reaction Mechanism. Food Chem. 2008, 108 (2), 779–783. 10.1016/j.foodchem.2007.11.022. 26059161

[ref47] Yodoshi M.; Ikuta T.; Mouri Y.; Suzuki S. Specific Extraction of Sialic-Acid-Containing Glycans and Glycopeptides Using Serotonin-Bonded Silica. Anal. Sci. 2010, 26 (1), 75–81. 10.2116/analsci.26.75. 20065591

[ref48] Woolley D. W.; Gommi B. W. Serotonin Receptors, VII. Activities of Various Pure Gangliosides as the Receptors. Proc. Natl. Acad. Sci. U.S.A. 1965, 53, 959–963. 10.1073/pnas.53.5.959. 5222564 PMC301355

[ref49] Traving C.; Schauer R. Structure, Function and Metabolism of Sialic Acids. Cell. Mol. Life Sci. 1998, 54 (12), 1330–1349. 10.1007/s000180050258. 9893709 PMC7082800

[ref50] Corfield A. P.; Winterburn P. J.; Clamp J. R.; et al. The Interaction of Sialic Acids with Immobilized 5-Hydroxytryptamine. Biochem. Soc. Trans. 1985, 13 (5), 956–957. 10.1042/bst0130956.

[ref51] Sturgeon R. J.; Sturgeon C. M. Affinity Chromatography of Sialoglycoproteins, Utilising the Interaction of Serotonin with N-Acetylneuraminic Acid and Its Derivatives. Carbohydr. Res. 1982, 103 (2), 213–219. 10.1016/s0008-6215(00)80684-9. 6180829

[ref52] Ochoa E. L. M.; Bangham A. D. N-Acetylneuraminic Acid Molecules As Possible Serotonin Binding Sites. J. Neurochem. 1976, 26 (6), 1193–1198. 10.1111/j.1471-4159.1976.tb07006.x. 58968

[ref53] Berry L. R.; Puzzuoli F. V.; Hatton M. W. C. On the Interaction between 5-Hydroxytryptamine and N-Acetylneuraminic Acid under Aqueous Conditions. Can. J. Biochem. Cell Biol. 1985, 63 (7), 757–763. 10.1139/o85-095. 4041969

[ref54] Pask-Hughes R. A. Characterization and Purification of Some Glycoproteins by High-Performance Liquid Chromatography. J. Chromatogr. A 1987, 393, 273–284. 10.1016/S0021-9673(01)94224-4. 3597603

[ref55] El Rassi Z.; Horváth C.; Yu R. K.; Ariga T. High-Performance Liquid Chromatography of Sialooligosaccharides and Gangliosides. J. Chromatogr. B 1989, 488 (1), 229–236. 10.1016/S0378-4347(00)82948-5. 2715282

[ref56] Naka R.; Kamoda S.; Ishizuka A.; Kinoshita M.; Kakehi K. Analysis of Total N-Glycans in Cell Membrane Fractions of Cancer Cells Using a Combination of Serotonin Affinity Chromatography and Normal Phase Chromatography. J. Proteome Res. 2006, 5 (1), 88–97. 10.1021/pr0502976. 16396498

[ref57] Meininger M.; Stepath M.; Hennig R.; Cajic S.; Rapp E.; Rotering H.; Wolff M. W.; Reichl U. Sialic Acid-Specific Affinity Chromatography for the Separation of Erythropoietin Glycoforms Using Serotonin as a Ligand. J. Chromatogr. B 2016, 1012–1013, 193–203. 10.1016/j.jchromb.2016.01.005. 26851523

[ref58] Lee C.; Gaston M. A.; Weiss A. A.; Zhang P. Colorimetric Viral Detection Based on Sialic Acid Stabilized Gold Nanoparticles. Biosens. Bioelectron. 2013, 42 (1), 236–241. 10.1016/j.bios.2012.10.067. 23208092 PMC3964789

[ref59] Song Z.; Duan C.; Shi M.; Li S.; Guan Y. One-Step Preparation of Zirconia Coated Silica Microspheres and Modification with D-Fructose 1, 6-Bisphosphate as Stationary Phase for Hydrophilic Interaction Chromatography. J. Chromatogr. A 2017, 1522, 30–37. 10.1016/j.chroma.2017.09.046. 28958759

[ref60] Rana R.; Rani S.; Kumar V.; Nakhate K. T.; Ajazuddin; Gupta U. Sialic Acid Conjugated Chitosan Nanoparticles: Modulation to Target Tumour Cells and Therapeutic Opportunities. AAPS PharmSciTech 2022, 23 (1), 10 10.1208/s12249-021-02170-z. 34862568

[ref61] Kumar K.; Govind S.; Mishra M.; Kumar A.; Chawla R.; et al. Dual Targeting PH Responsive Chitosan Nanoparticles for Enhanced Active Cellular Internalization of Gemcitabine in Non-Small Cell Lung Cancer. Int. J. Biol. Macromol. 2023, 249, 126057 10.1016/j.ijbiomac.2023.126057. 37524283

[ref62] Pratuangdejkul J.; Nosoongnoen W.; Guérin G. A.; Loric S.; Conti M.; Launay J. M.; Manivet P. Conformational Dependence of Serotonin Theoretical PKa Prediction. Chem. Phys. Lett. 2006, 420 (4–6), 538–544. 10.1016/j.cplett.2006.01.035.

[ref63] Vimr E. R.; Kalivoda K. A.; Deszo E. L.; Steenbergen S. M. Diversity of Microbial Sialic Acid Metabolism. Microbiol. Mol. Biol. Rev. 2004, 68 (1), 132–153. 10.1128/MMBR.68.1.132-153.2004. 15007099 PMC362108

[ref64] Xiong Y.; Liu X.; Xiong H. Aggregation Modeling of the Influence of PH on the Aggregation of Variably Charged Nanoparticles. Sci. Rep. 2021, 11 (1), 17386 10.1038/s41598-021-96798-3. 34462496 PMC8405828

[ref65] Scheepers M. R. W.; Haenen S. R. R.; Coers J. M.; Van Ijzendoorn L. J.; Prins M. W. J. Inter-Particle Biomolecular Reactivity Tuned by Surface Crowders. Nanoscale 2020, 12 (27), 14605–14614. 10.1039/D0NR03125A. 32614022

[ref66] Tuchtenhagen M.; Stiboller M.; Witt B.; Schwerdtle T. A Novel Approach for the Determination of Exchangeable Copper in Serum Using Protein Precipitation. J. Anal. At. Spectrom. 2023, 38 (3), 587–594. 10.1039/D2JA00355D.

[ref67] Carmical J.; Brown S. The Impact of Phospholipids and Phospholipid Removal on Bioanalytical Method Performance. Biomed. Chromatogr. 2016, 30 (5), 710–720. 10.1002/bmc.3686. 26773720

[ref68] Xu J.; Wu P.; Ye E. C.; Yuan B. F.; Feng Y. Q. Metal Oxides in Sample Pretreatment. TrAC, Trends Anal. Chem. 2016, 80, 41–56. 10.1016/j.trac.2016.02.027.

