# Supplementary material for: Sialic Acid-Functionalized Gold Nanoparticles for Sensitive and Selective Colorimetric Determination of Serotonin
Source: ACS Omega. 2024 May 23;9(22):23832–42. doi: 10.1021/acsomega.4c01859 (PMC11154895; doi:10.1021/acsomega.4c01859)
Supplement: Supplementary file 1 — ao4c01859_si_001.pdf [file ao4c01859_si_001.pdf]

Supporting Information

Sialic Acid-Functionalized Gold Nanoparticles for Sensitive and Selective Colorimetric Determination of Serotonin

Begüm Avcı[1], Yeliz Akpınar[2], Gülay Ertaş[1], Mürvet Volkan[1*]

[1]Department of Chemistry, Middle East Technical University, 06800, Ankara, Turkey

[2]Department of Chemistry, Kirsehir Ahi Evran University, 40100, Kirsehir, Turkey

*E-mail: murvet@metu.edu.tr

Experimental Setup for The Column

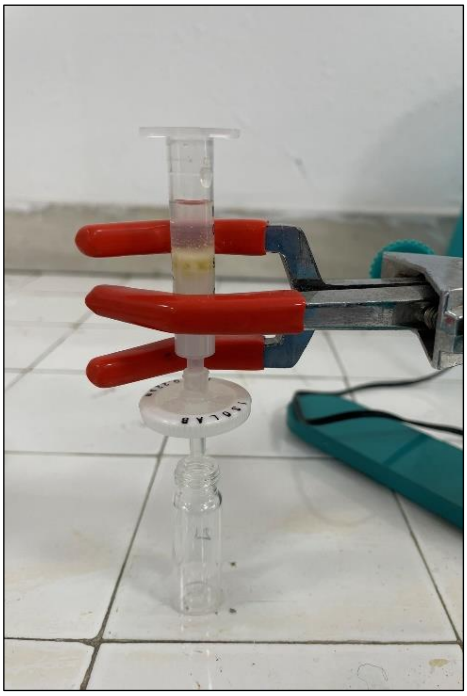

Figure S1. Setup for ZrO_2/SiO_2 column.

UV-vis Absorption Spectrum of SA-AuNPs

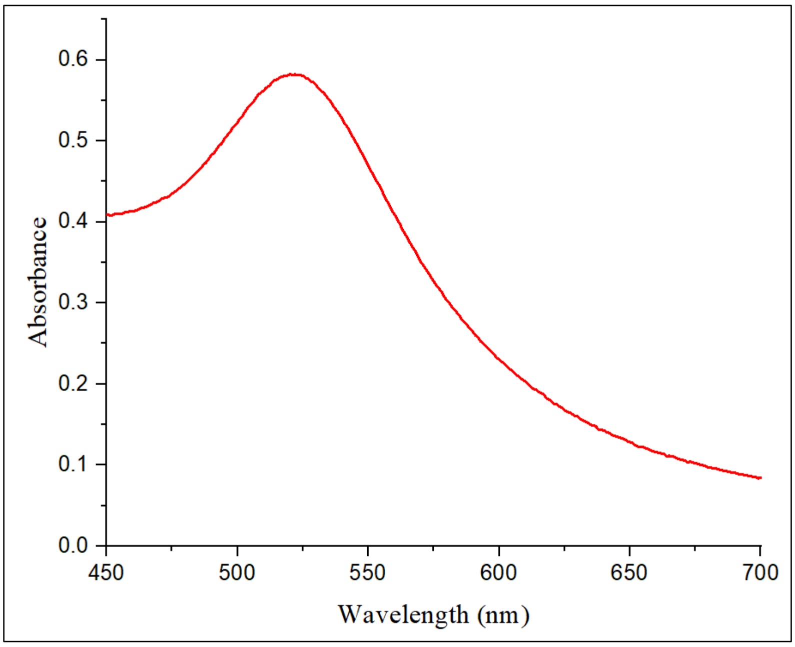

Figure S2. The UV-vis absorption spectrum of SA-AuNPs (sialic acid-functionalized gold nanoparticles) at the optimum condition.

The surface plasmon resonance (SPR) peak of SA-AuNPs appears around 520 nm, which corresponds to the wine-red color of the nanoparticles.

FT-IR Spectrum of Sialic Acid

FT-IR spectrum of sialic acid (SA) was taken in the range of 400-4000 cm^{-1}. Sialic acid has five hydroxyl groups, one *N*-acetyl group, and one carboxyl group. The sialic acid vibrational bands similar to the spectrum of sialic acid stabilized AuNPs yielded at 3341, 2934, 1656, 1438, and 1374 cm^{-1} as seen in Figure S3, corresponding to O-H stretching, C-H stretching, N-H bending, C-H bending, and O-H bending bands, respectively.

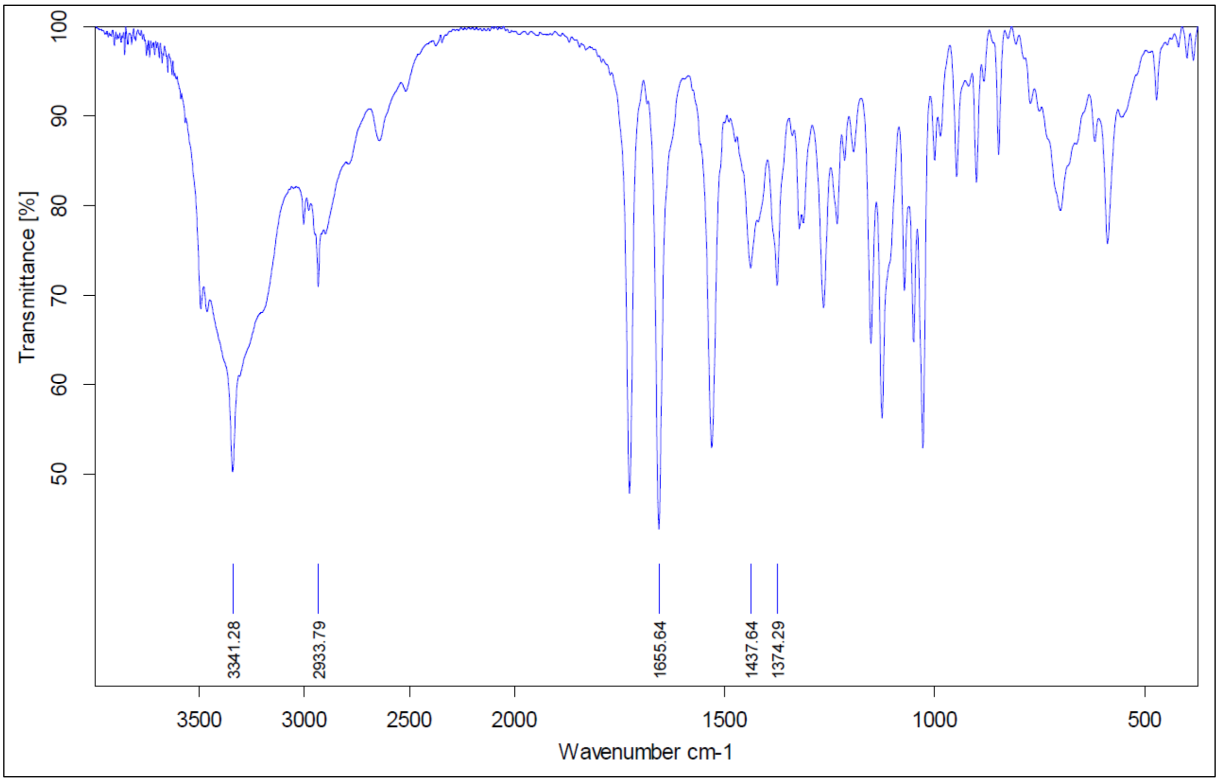

Figure S3. FT-IR spectrum of solid sialic acid (SA).

Effect of pH

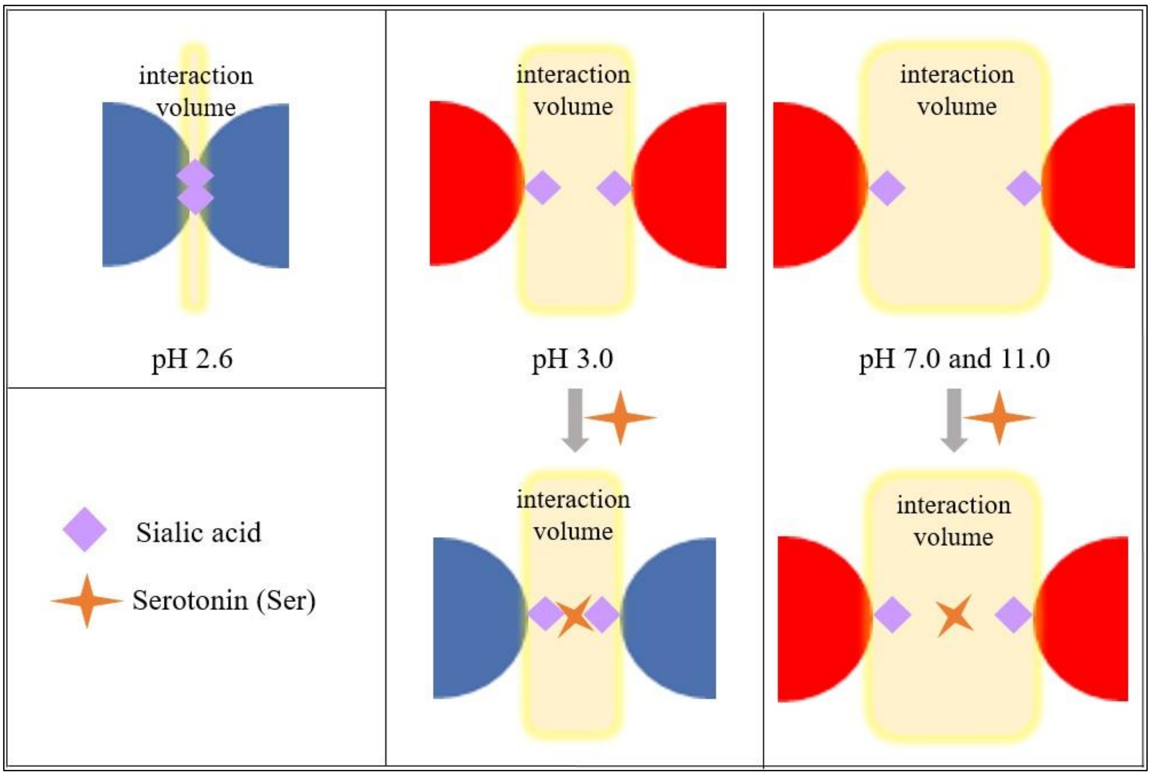

Figure S4. The change in the interaction volume depending on the pH of the medium.

Colorimetric Determination of Serotonin in the BSA Matrix

Table S1. Results for the Determination of Serotonin in the Surrogate Matrix

	**Spiked Amount (µM)**	**Found Amount (µM)***	**Recovery (%)***
**BSA Surrogate Matrix**	0.244	0.235 ± 0.009	96.2 ± 3.5
	0.488	0.498 ± 0.003	102.1 ± 0.8
	0.793	0.831 ± 0.033	104.8 ± 4.2
*** mean ± standard deviation, n=3**			
